# Supplementary material for: Pacific subduction control on Asian continental deformation including Tibetan extension and eastward extrusion tectonics
Source: Nat Commun. 2019 Oct 2;10:4480. doi: 10.1038/s41467-019-12337-9 (PMC6775058; doi:10.1038/s41467-019-12337-9)
Supplement: Supplementary file 1 — Supplementary Information [file 41467_2019_12337_MOESM1_ESM.pdf]

Supplementary Information

Pacific subduction control on Asian continental deformation including Tibetan extension and eastward extrusion tectonics

Schellart et al.

Supplementary Note 1

Structural-tectonic map Central and East and Southeast Asia

The structural and tectonic map of Central, East and Southeast Asia and surrounding regions as shown in Fig. 1a is compiled, simplified and modified from many previous works. These include several references for the entire region displayed on the map[1-3] and the Himalaya-Tibetan region[4]. For several individual basins or local structures the references are as follows, from north to south: Kamchatka grabens[5], Shantar-Lizianski basin[6], Priokhotsky rift[7], Sea of Okhotsk[6,8], Heilongjiang basin[9], Bohai basin[10], Northern Yellow Sea basin[11], South Yellow Sea basin[12], Subei basin[12], Gunsan basin[12], Tibet rifts[4,13-15], East China Sea basin[16], Yuanma basin[17], Taiwan Strait basins[18], South China Sea margins[19], Andaman Sea[20-21], Mergui basin[20-21], Malay basin[19-21], Sumatra basins[22,19], East Borneo basins[22], Java basins[22].

The timing of extension for the individual basins/extensional structures as shown in Fig. 1a is deduced from the following references: Sea of Okhotsk[6], Kamchatka grabens[5], Kuril basin[23-24], Shantar-Liziansky basin[6], Priokhotsky rift[7], Baikal rift[25], Heilongjiang basin[9], Sea of Japan[26], Sea of Japan margins[27-29], Yilan Yitong graben[30], Hetao-Yinchuan grabens[31], South Ningxia region[32], Shanxi graben[33], Bohai basin[3,34], North Yellow Sea basin[11], northern South Yellow Sea basin and Gunsan basin[12,35], Subei basin and southern South Yellow Sea basin[36], southern North China basin[3], Weihe graben[31], north-south trending Tibetan rifts and dikes[4,37-38], Jianghan basin[39], Yuanma basin[17], East China Sea basins[16], Okinawa Trough[40], Taiwan Strait basins[18], Pearl River Mouth basins[41], Beibuwan basin[42], Yinggehai-Song Hong basin[43], Quiondongnan basin[41], Phu Khan basin[44], Nam Con Son basin[44], South China Sea[45], southern South China Sea[46], Pattani basin[21], Mergui basin[20], Andaman Sea[20], Malay basin[47], Sumatra basins[22], Java basins[22], East Borneo basins[22], Sulu Sea[48-50], Celebes Sea[48-50], Gorontalo basins[51], Banda Sea[52].

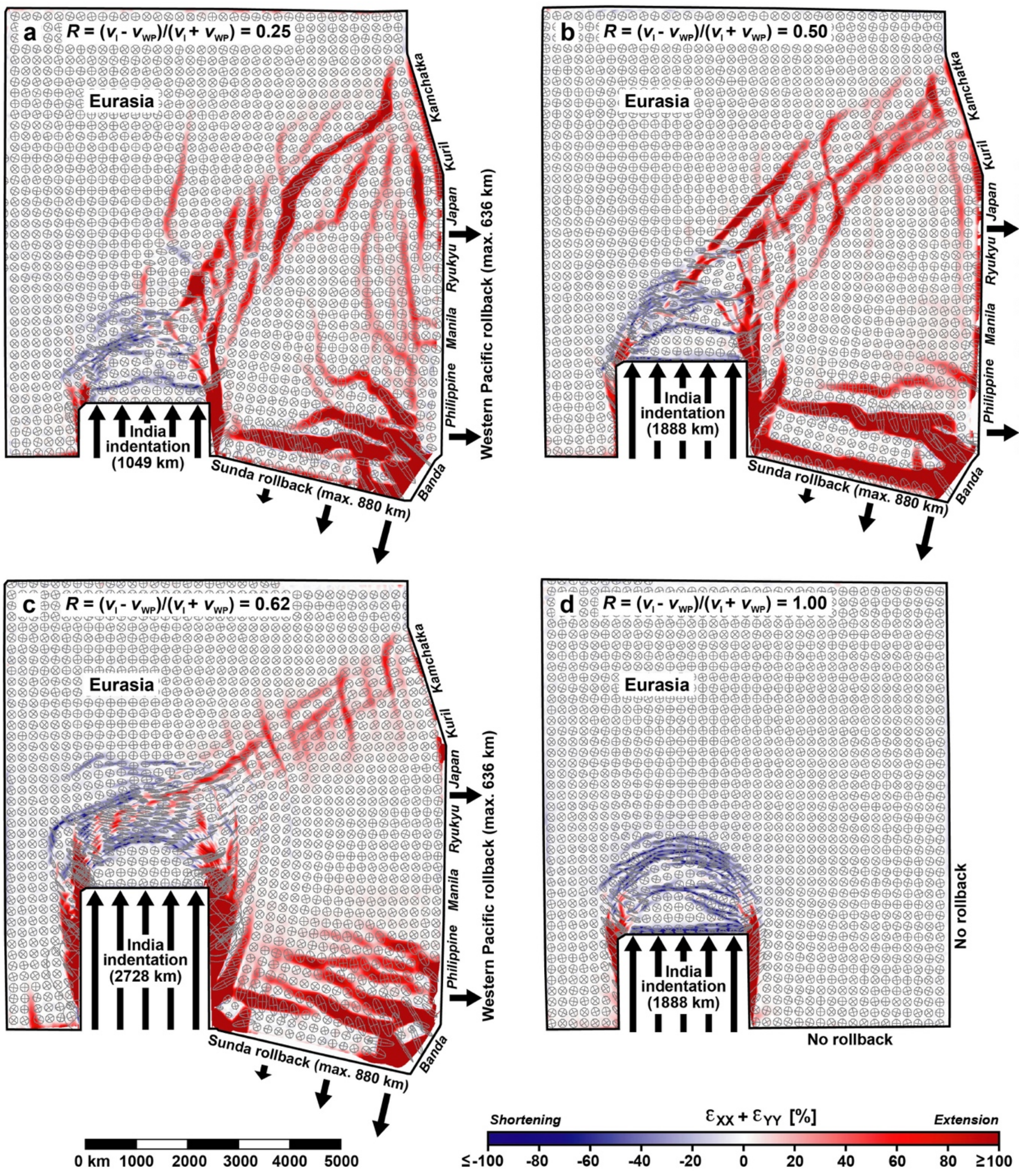

Supplementary Fig. 1 | Top views of four experiments with different velocity ratios showing finite horizontal normal strain ($\varepsilon_{XX} + \varepsilon_{YY}$) and the horizontal finite strain ellipse field. The experiments simulate Asian continental deformation and the velocity ratio is expressed as $R = (v_I - v_{WP})/(v_I + v_{WP})$, where v_I = Indian continental subduction hinge and slab advance (roll-forward) velocity and v_{WP} = Pacific subduction hinge and slab retreat (rollback) velocity. The results are shown for the end of each experimental run. **a**, Experiment I_{MIN}-R with R = 0.25 (minimum indentation). **b**, Experiment I_{INT}-R with R = 0.50 (intermediate indentation). **c**, Experiment I_{MAX}-R with R = 0.62 (maximum indentation). **d**, Experiment I_{INT}-NR with R = 1.00 (intermediate indentation and no rollback).

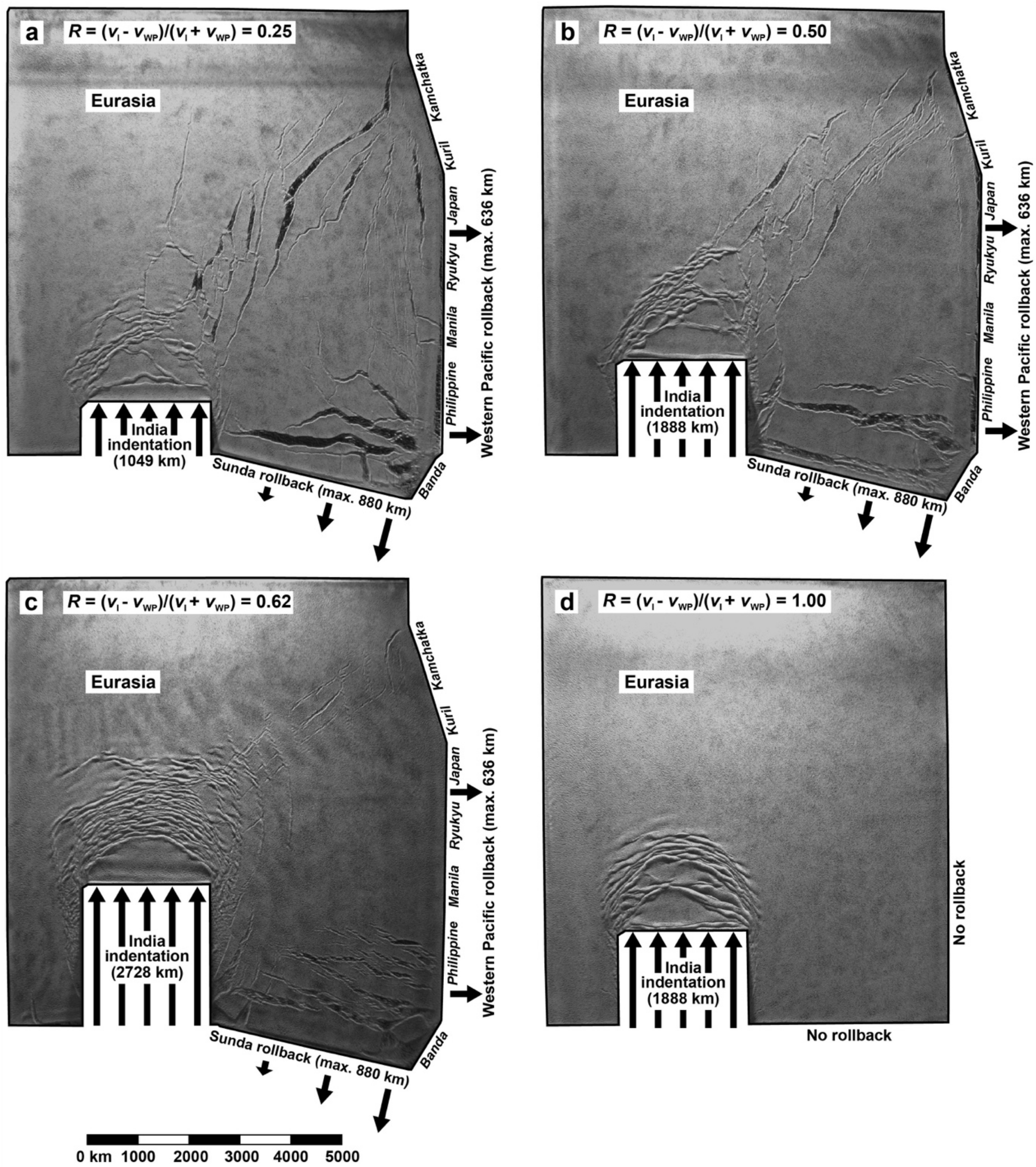

Supplementary Fig. 2. | Top views of four experiments simulating Asian deformation with different velocity ratios (*R*) showing the digital photographs. The results are shown for the end of each experimental run. **a**, Experiment I_{MIN}-R with $R = 0.25$ (minimum indentation). **b**, Experiment I_{INT}-R with $R = 0.50$ (intermediate indentation). **c**, Experiment I_{MAX}-R with $R = 0.62$ (maximum indentation). **d**, Experiment I_{INT}-NR with $R = 1.00$ (intermediate indentation and no rollback). Low-angle lighting is from the north.

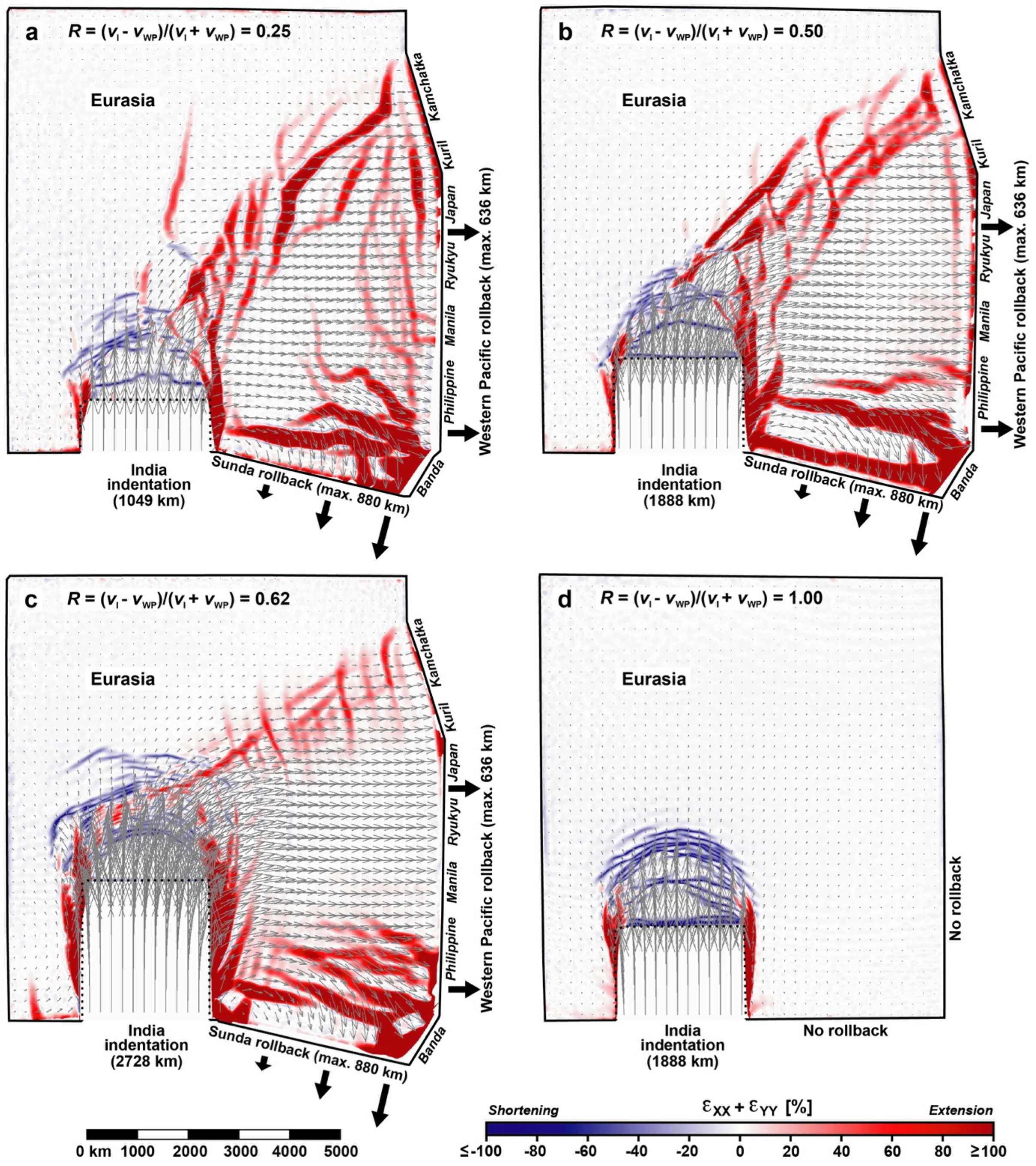

Supplementary Fig. 3 | Top views of four experiments simulating Asian deformation with different velocity ratios (*R*) showing finite horizontal normal strain ($\varepsilon_{XX} + \varepsilon_{YY}$) and the horizontal finite displacement field. The results are shown for the end of each experimental run. **a**, Experiment I_{MIN}-R with $R = 0.25$ (minimum indentation). **b**, Experiment I_{INT}-R with $R = 0.50$ (intermediate indentation). **c**, Experiment I_{MAX}-R with $R = 0.62$ (maximum indentation). **d**, Experiment I_{INT}-NR with $R = 1.00$ (intermediate indentation and no rollback).

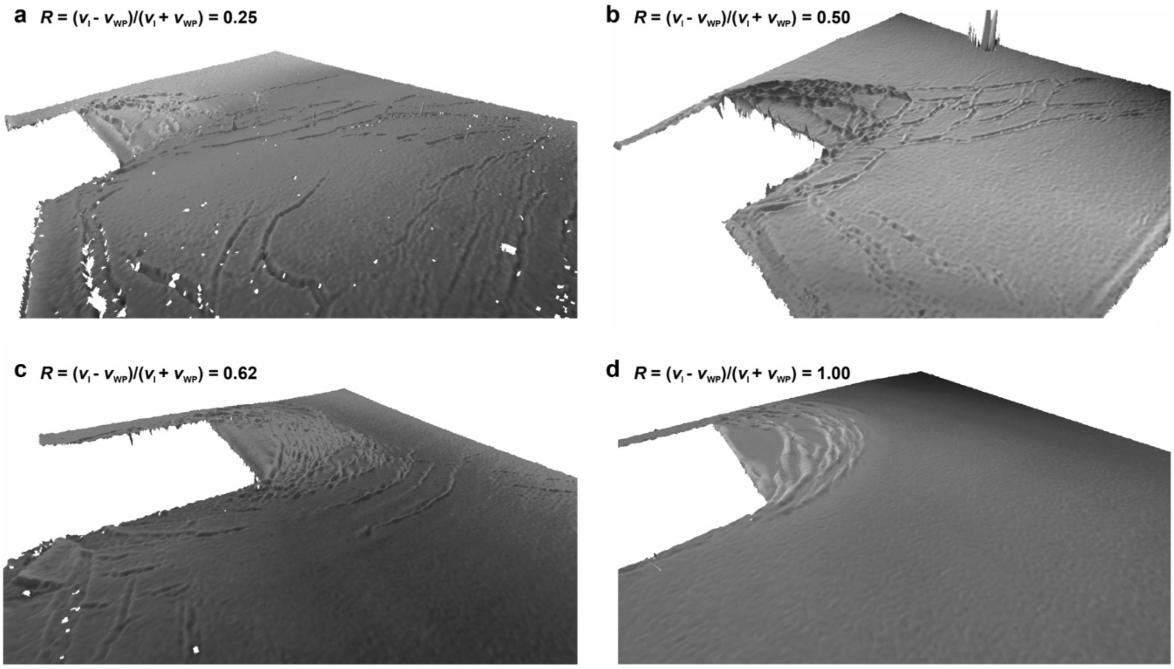

Supplementary Fig. 4 | Three-dimensional perspective views of four experiments simulating Asian deformation with different velocity ratios $R = (v_I - v_{WP})/(v_I + v_{WP})$ showing the surface topography. The results are shown for the end of each experimental run. **a**, Experiment I_{MIN}-R with $R = 0.25$ (minimum indentation). The maximum topography difference between mountain ranges in the experimental Himalaya-Tibet region and undeformed foreland is 1.0-1.9 mm, scaling to 3.5-6.7 km in nature (using the scaling as described in Table 2). **b**, Experiment I_{INT}-R with $R = 0.50$ (intermediate indentation). The maximum topography difference between mountain ranges in the experimental Himalaya-Tibet region and undeformed foreland is 1.5-2.6 mm, scaling to 5.3-9.2 km in nature. **c**, Experiment I_{MAX}-R with $R = 0.62$ (maximum indentation). The maximum topography difference between mountain ranges in the experimental Himalaya-Tibet region and undeformed foreland is 2.8-4.0 mm, scaling to 9.9-14.1 km in nature. **d**, Experiment I_{INT}-NR with $R = 1.00$ (intermediate indentation and no rollback). The maximum topography difference between mountain ranges in the experimental Himalaya-Tibet region and undeformed foreland is 4-5.2 mm, scaling to 14.1-18.3 km in nature. The maximum topography values for experiments I_{MIN}-R and I_{INT}-R in **a** and **b** are reasonable and comparable to values in nature (Tibetan Plateau at ~5 km elevation and mountains up to 8-9 km high), the values for experiments I_{MAX}-R and I_{INT}-NR in **c** and **d** are higher than the values in nature.

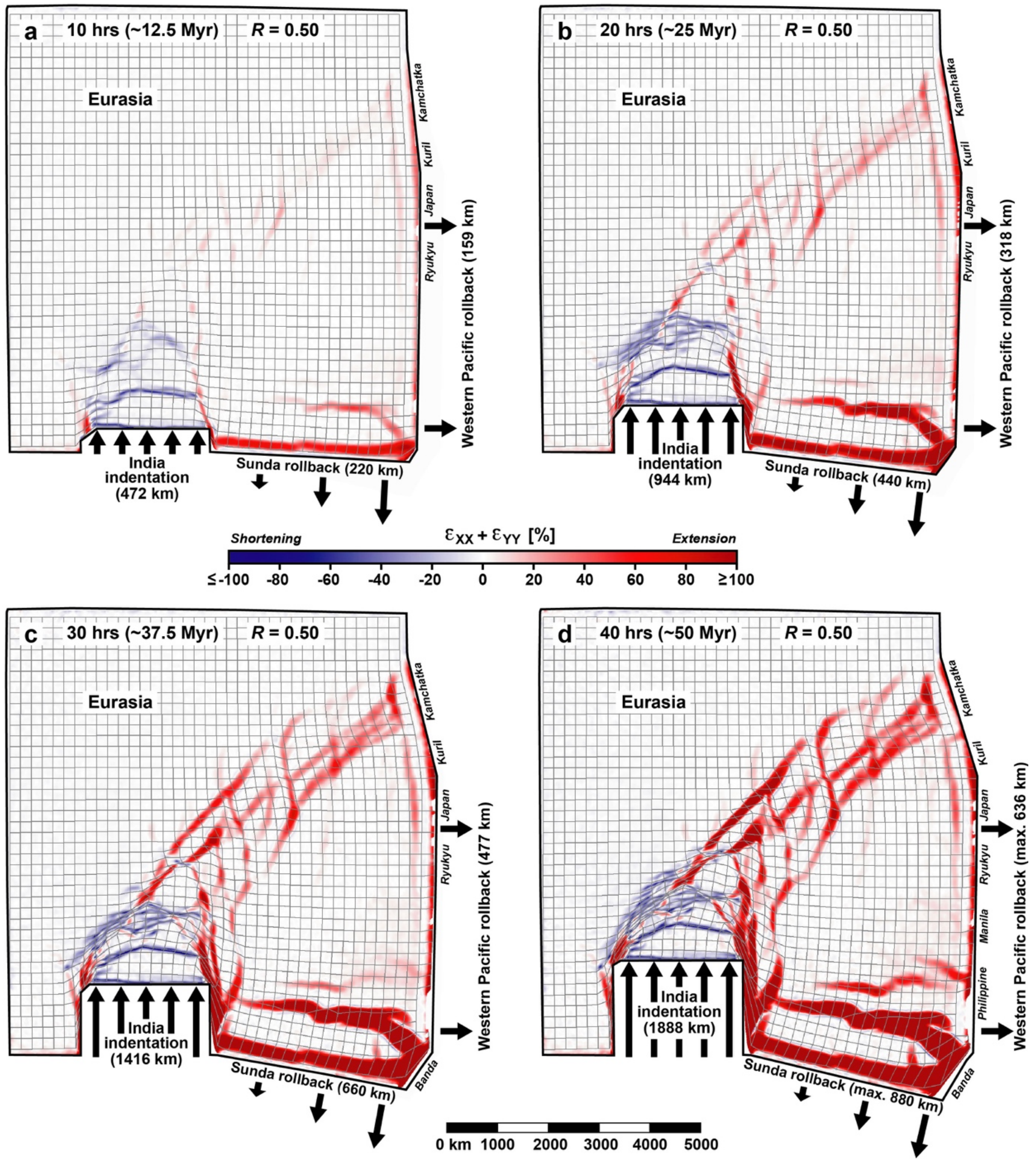

Supplementary Fig. 5 | Top views of experiment I_{INT}-R (with *R* = 0.50, intermediate indentation) showing the finite horizontal normal strain ($\varepsilon_{XX} + \varepsilon_{YY}$) and deformed model grid at four different times during the evolution of the model. a, 10 hours corresponding to ~12.5 Myr (~middle-late Eocene). **b**, 20 hours corresponding to ~25 Myr (~late Oligocene). **c**, 30 hours corresponding to ~37.5 Myr (~middle Miocene). **d**, 40 hours corresponding to ~50 Myr (~present).

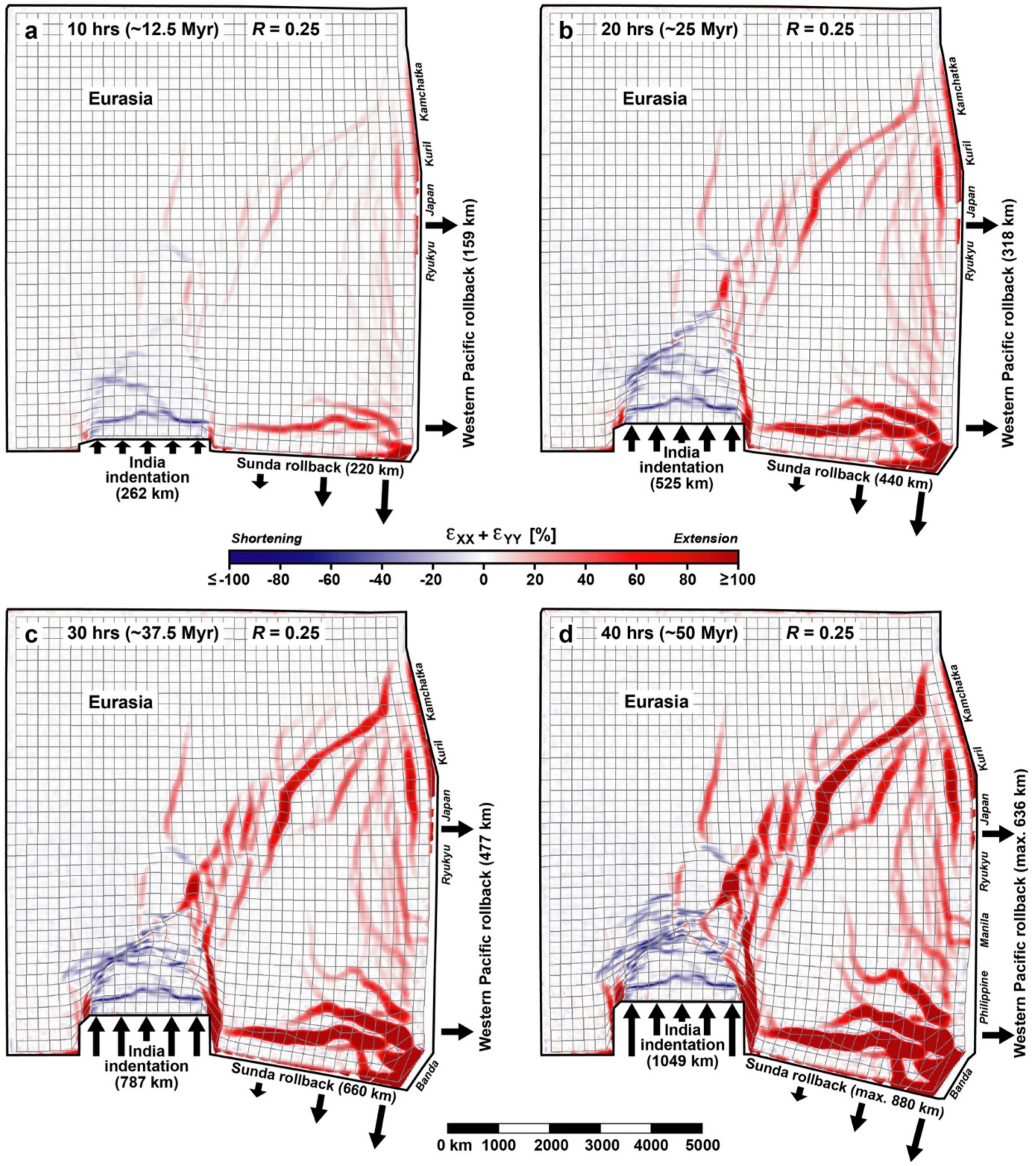

Supplementary Fig. 6 | Top views of experiment I_{MIN}-R (with R = 0.25, minimum indentation) showing the finite horizontal normal strain ($\varepsilon_{XX} + \varepsilon_{YY}$) and deformed model grid at four different times during the evolution of the model. **a**, 10 hours corresponding to ~12.5 Myr (~middle-late Eocene). **b**, 20 hours corresponding to ~25 Myr (~late Oligocene). **c**, 30 hours corresponding to ~37.5 Myr (~middle Miocene). **d**, 40 hours corresponding to ~50 Myr (~present).

Supplementary References

1. Tapponnier, R., Peltzer, G., Le Dain, A. Y., Armijo, R. & Cobbold, P. Propagating extrusion tectonics in Asia; new insights from simple experiments with plasticine. *Geology* **10**, 611-616 (1982).
2. Schellart, W. P. & Lister, G. S. The role of the East Asian active margin in widespread extensional and strike-slip deformation in East Asia. *Journal of the Geological Society, London* **162**, 959-972 (2005).
3. Ren, J., Tamaki, K., Sitian, L. & Junxia, Z. Late Mesozoic and Cenozoic rifting and its dynamic setting in Eastern China and adjacent areas. *Tectonophysics* **344**, 175-205 (2002).
4. Li, Y. *et al.* Propagation of the deformation and growth of the Tibetan–Himalayan orogen: A review. *Earth-Science Reviews*, 36-61, doi:10.1016/j.earscirev.2015.01.001 (2015).
5. Kozhurin, A. & Zelenin, E. An extending island arc: The case of Kamchatka. *Tectonophysics* **706-707**, 91-102, doi:10.1016/j.tecto.2017.04.001 (2017).
6. Worrall, D. M., Kruglyak, V., Kunst, F. & Kuznetsov, V. Tertiary tectonics of the Sea of Okhotsk, Russia: Far-field effects of the India-Eurasia collision. *Tectonics* **15**, 813-826 (1996).
7. Zabrodin, V. Y. Tectonics and Evolution of the Northeastern Extremity of the East-Asian Rift Belt. *Russian Journal of Pacific Geology* **11**, 155-162 (2017).
8. Schellart, W. P., Jessell, M. W. & Lister, G. S. Asymmetric deformation in the backarc region of the Kuril arc, northwest Pacific: New insights from analogue modeling. *Tectonics* **22 (5)**, 1047, doi:1010.1029/2002TC001473 (2003).
9. Zhao, X. *et al.* Characteristics, structural styles and tectonic implications of Mesozoic-Cenozoic faults in the eastern Heilongjiang basins (NE China). *Journal of Asian Earth Sciences* **146**, 196-210, doi:10.1016/j.jseaes.2017.05.004 (2017).
10. Zhou, J. & Zhou, J. Mechanisms of Cenozoic deformation in the Bohai Basin, Northeast China: Physical modelling and discussions. *Science in China: Series D Earth Sciences* **49**, 258-271, doi:10.1007/s11430-006-0258-z (2006).
11. Li, W., Lu, W., Liu, Y. & Xu, J. Superimposed versus residual basin: The North Yellow Sea Basin. *Geoscience Frontiers* **3**, 33-39, doi:10.1016/j.gsf.2011.11.001 (2012).
12. Shinn, Y. J., Chough, S. K. & Hwang, I. G. Structural development and tectonic evolution of Gunsan Basin (Cretaceous–Tertiary) in the central Yellow Sea. *Marine and Petroleum Geology* **27**, 500-514, doi:10.1016/j.marpetgeo.2009.11.001 (2010).
13. Tapponnier, P. *et al.* Oblique Stepwise Rise and Growth of the Tibet Plateau. *Science* **294**, 1671-1677 (2001).
14. Searle, M. P., Elliott, J. R., Phillips, R. J. & Chung, S.-L. Crustal–lithospheric structure and continental extrusion of Tibet. *Journal of the Geological Society, London* **168**, 633-672, doi:10.1144/0016-76492010-139 (2011).
15. Ratschbacher, L. *et al.* Rifting and strike–slip shear in central Tibet and the geometry, age and kinematics of upper crustal extension in Tibet. *Geological Society, London, Special Publications* **353**, 127-163, doi:10.1144/SP353.8 (2011).
16. Cukur, D., Horozal, S., Kim, D. C. & Han, H. C. Seismic stratigraphy and structural analysis of the northern East China Sea Shelf Basin interpreted from multi-channel seismic reflection data and cross-section restoration. *Marine and Petroleum Geology* **28**, 1003-1022, doi:10.1016/j.marpetgeo.2011.01.002 (2011).
17. Li, J., Zhang, Y., Dong, S. & Li, H. Late Mesozoic–Early Cenozoic deformation history of the Yuanma Basin, central South China. *Tectonophysics* **570-571**, 163-183, doi:10.1016/j.tecto.2012.08.012 (2012).
18. Huang, C.-Y., Shea, K.-H. & Li, Q. A foraminiferal study on Middle Eocene-Oligocene break-up unconformity in northern Taiwan and its correlation with IODP Site U1435 to constrain the onset event of South China Sea opening. *Journal of Asian Earth Sciences* **138**, 439-465, doi:10.1016/j.jseaes.2016.09.014 (2017).
19. Pubellier, M. & Morley, C. K. The basins of Sundaland (SE Asia): Evolution and boundary conditions. *Marine and Petroleum Geology* **58**, 555-578, doi:10.1016/j.marpetgeo.2013.11.019 (2014).
20. Curray, J. R. Tectonics and history of the Andaman Sea region. *Journal of Asian Earth Sciences*

25, 187-232, doi:10.1016/j.jseaes.2004.09.001 (2005).
21. Sautter, B. *et al.* Late Paleogene rifting along the Malay Peninsula thickened crust. *Tectonophysics* **710-711**, 205-224, doi:10.1016/j.tecto.2016.11.035 (2017).
22. Doust, H. & Noble, R. A. Petroleum systems of Indonesia. *Marine and Petroleum Geology* **25**, 103-129, doi:10.1016/j.marpetgeo.2007.05.007 (2008).
23. Takeuchi, T., Kodama, K. & Ozawa, T. Paleomagnetic evidence for block rotations in central Hokkaido-south Sakhalin, Northeast Asia. *Earth and Planetary Science Letters* **169**, 7-21 (1999).
24. Ikeda, Y., Stern, R. J., Kagami, H. & Sun, C.-H. Pb, Nd, and Sr isotopic constraints on the origin of Miocene basaltic rocks from northeast Hokkaido, Japan: Implications for opening of the Kurile back-arc basin. *The Island Arc* **9**, 161-172 (2000).
25. Ivanov, A. V. *et al.* Volcanism in the Baikal rift: 40 years of active-versus-passive model discussion. *Earth-Science Reviews* **148**, 18-43, doi:10.1016/j.earscirev.2015.05.011 (2015).
26. Nohda, S. Formation of the Japan Sea basin: Reassessment from Ar–Ar ages and Nd–Sr isotopic data of basement basalts of the Japan Sea and adjacent regions. *Journal of Asian Earth Sciences* **34**, 599-609, doi:10.1016/j.jseaes.2008.08.003 (2009).
27. Yoon, S.-H., Sohn, Y. K. & Chough, S. K. Tectonic, sedimentary, and volcanic evolution of a back-arc basin in the East Sea (Sea of Japan). *Marine Geology* **352**, 70-88, doi:10.1016/j.margeo.2014.03.004 (2014).
28. Son, M. *et al.* Miocene tectonic evolution of the basins and fault systems, SE Korea: dextral, simple shear during the East Sea (Sea of Japan) opening. *Journal of the Geological Society, London* **172**, 664-680, doi:10.1144/jgs2014-079 (2015).
29. Ishiyama, T. *et al.* Structures and active tectonics of compressionally reactivated back-arc failed rift across the Toyama trough in the Sea of Japan, revealed by multiscale seismic profiling. *Tectonophysics* **710-711**, 21-36, doi:10.1016/j.tecto.2016.09.029 (2017).
30. Gu, C. *et al.* Cenozoic evolution of the Yilan–Yitong Graben in NE China: An example of graben formation controlled by pre-existing structures. *Journal of Asian Earth Sciences* **146**, 168-184, doi:10.1016/j.jseaes.2017.05.024 (2017).
31. Zhang, Y. Q., Mercier, J. L. & Vergély, P. Extension in the graben systems around the Ordos (China), and its contribution to the extrusion tectonics of south China with respect to Gobi-Mongolia. *Tectonophysics* **285**, 41-75 (1998).
32. Shi, W. *et al.* Cenozoic tectonic evolution of the South Ningxia region, northeastern Tibetan Plateau inferred from new structural investigations and fault kinematic analyses. *Tectonophysics* **649**, 139-164, doi:10.1016/j.tecto.2015.02.024 (2015).
33. Shi, W. *et al.* Evolution of the late Cenozoic tectonic stress regime in the Shanxi Rift, central North China Plate inferred from new fault kinematic analysis. *Journal of Asian Earth Sciences* **114**, 54-72, doi:10.1016/j.jseaes.2015.04.044 (2015).
34. Luo, L. *et al.* Geometry and evolution of the Cangdong sag in the Bohai Bay basin, China: Implications for subduction of the Pacific plate. *Scientific Reports* **7**, 15393, doi:10.1038/s41598-017-15759-x (2017).
35. Shinn, Y. J. Geological structures and controls on half-graben inversion in the western Gunsan Basin, Yellow Sea. *Marine and Petroleum Geology* **68**, 480-491, doi:10.1016/j.marpetgeo.2015.09.013 (2015).
36. Liu, Y. *et al.* Influence of normal fault growth and linkage on the evolution of a rift basin: A case from the Gaoyou depression of the Subei Basin, eastern China. *AAPG Bulletin* **101**, 265-288, doi:10.1306/06281615008 (2017).
37. Mitsuishi, M., Wallis, S. R., Aoya, M., Lee, J. & Wang, Y. E–W extension at 19 Ma in the Kung Co area, S. Tibet: Evidence for contemporaneous E–W and N–S extension in the Himalayan orogen. *Earth and Planetary Science Letters* **325-326**, 10-20, doi:10.1016/j.epsl.2011.11.013 (2012).
38. Wang, Q. *et al.* Eocene north–south trending dikes in central Tibet: New constraints on the timing of east–west extension with implications for early plateau uplift? *Earth and Planetary Science Letters* **298**, 205-216, doi:10.1016/j.epsl.2010.07.046 (2010).
39. Huang, C. & Hinnov, L. Evolution of an Eocene–Oligocene saline lake depositional system and its controlling factors, Jianghan Basin, China. *Journal of Earth Science* **25**, 959-976, doi:10.1007/s12583-014-0499-2 (2014).

40. Shang, L.-N. *et al.* Late Cenozoic evolution of the East China continental margin: Insights from seismic, gravity, and magnetic analyses. *Tectonophysics* **698**, 1-15, doi:10.1016/j.tecto.2017.01.003 (2017).
41. Wu, Z., Zhu, W., Shao, L. & Xu, C. Sedimentary facies and the rifting process during the late Cretaceous to early Oligocene in the northern continental margin, South China Sea. *Interpretation* **4**, SP33-SP45, doi:10.1190/INT-2015-0163.1 (2016).
42. Liu, E. *et al.* Sedimentary characteristics and tectonic setting of sublacustrine fans in a half-graben rift depression, Beibuwan Basin, South China Sea. *Marine and Petroleum Geology* **52**, 9-21, doi:10.1016/j.marpetgeo.2014.01.008 (2014).
43. Clift, P. D. & Sun, Z. The sedimentary and tectonic evolution of the Yinggehai–Song Hong basin and the southern Hainan margin, South China Sea: Implications for Tibetan uplift and monsoon intensification. *Journal of Geophysical Research, B, Solid Earth and Planets* **111**, B06405, doi:10.1029/2005JB004048 (2006).
44. Morley, C. K. Major unconformities/termination of extension events and associated surfaces in the South China Seas: Review and implications for tectonic development. *Journal of Asian Earth Sciences* **120**, 62-86, doi:10.1016/j.jseaes.2016.01.013 (2016).
45. Li, C.-F. *et al.* Ages and magnetic structures of the South China Sea constrained by deep tow magnetic surveys and IODP Expedition 349. *Geochemistry Geophysics Geosystems* **15**, 4958-4983, doi:10.1002/2014GC005567 (2014).
46. Yang, M. *et al.* Petroleum systems of the major sedimentary basins in Nansha sea waters (South China Sea). *Earth Science Frontiers* **22**, 48-58, doi:10.13745/j.esf.2015.03.004 (2015).
47. Fyhn, M. B. W., Boldreel, L. O. & Nielsen, L. H. Escape tectonism in the Gulf of Thailand: Paleogene left-lateral pull-apart rifting in the Vietnamese part of the Malay Basin. *Tectonophysics* **483**, 365-376, doi:10.1016/j.tecto.2009.11.004 (2010).
48. Silver, E. A. & Rangin, C. Leg 124 Tectonic synthesis. *Proceedings of the Ocean Drilling Program, Scientific Results* **124**, 3-9 (1991).
49. Rangin, C. & Silver, E. A. Neogene tectonic evolution of the Celebes-Sulu basins: New insights from Leg 124 drilling. *Proceedings of the Ocean Drilling Program, Scientific Results* **124**, 51-63 (1991).
50. Hall, R. Cenozoic geological and plate tectonic evolution of SE Asia and the SW Pacific; computer-based reconstructions, model and animations. *Journal of Asian Earth Sciences* **20**, 353-431 (2002).
51. Pezzati, G., Hall, R., Burgess, P. & Perez-Gussinye, M. The Poso basin in Gorontalo Bay, Sulawesi: Extension related to core complex formation on land. *Proceedings, Indonesian Petroleum Association*, IPA14-G-297 (2014).
52. Spakman, W. & Hall, R. Surface deformation and slab–mantle interaction during Banda arc subduction rollback. *Nature Geoscience* **3**, 562-566, doi:10.1038/NGEO917 (2010).
